# Unveiling the principle descriptor for predicting the electron inelastic mean free path based on a machine learning framework

**DOI:** 10.1080/14686996.2019.1689785

**Published:** 2019-11-07

**Authors:** Xun Liu, Zhufeng Hou, Dabao Lu, Bo Da, Hideki Yoshikawa, Shigeo Tanuma, Yang Sun, Zejun Ding

**Affiliations:** a Hefei National Laboratory for Physical Sciences at Microscale and Department of Physics, University of Science and Technology of China, Hefei, Anhui, People’s Republic of China; b Research and Services Division of Materials Data and Integrated System, National Institute for Materials Science, Tsukuba, Ibaraki, Japan; c Research Center for Advanced Measurement and Characterization, National Institute for Materials Science, Tsukuba, Ibaraki, Japan; d State Key Laboratory of Structural Chemistry, Fujian Institute of Research on the Structure of Matter, Chinese Academy of Sciences, Fuzhou, China; e US Department of Energy, Ames Laboratory, Ames, IA, USA

**Keywords:** Surface science, machine learning, inelastic mean free path, the Least Absolute Shrinkage and Selection Operator (LASSO), 212 Surface and interfaces, 404 Materials informatics / Genomics

## Abstract

The TPP-2M formula is the most popular empirical formula for the estimation of the electron inelastic mean free paths (IMFPs) in solids from several simple material parameters. The TPP-2M formula, however, poorly describes several materials because it relies heavily on the traditional least-squares analysis. Herein, we propose a new framework based on machine learning to overcome the weakness. This framework allows a selection from an enormous number of combined terms (descriptors) to build a new formula that describes the electron IMFPs. The resulting framework not only provides higher average accuracy and stability but also reveals the physics meanings of several newly found descriptors. Using the identified principle descriptors, a complete physics picture of electron IMFPs is obtained, including both single and collective electron behaviors of inelastic scattering. Our findings suggest that machine learning is robust and efficient to predict the IMFP and has great potential in building a regression framework for data-driven problems. Furthermore, this method could be applicable to find empirical formula for given experimental data using a series of parameters given a priori, holds potential to find a deeper connection between experimental data and a priori parameters.

## GRAPHICAL ABSTRACT

**Figure:**
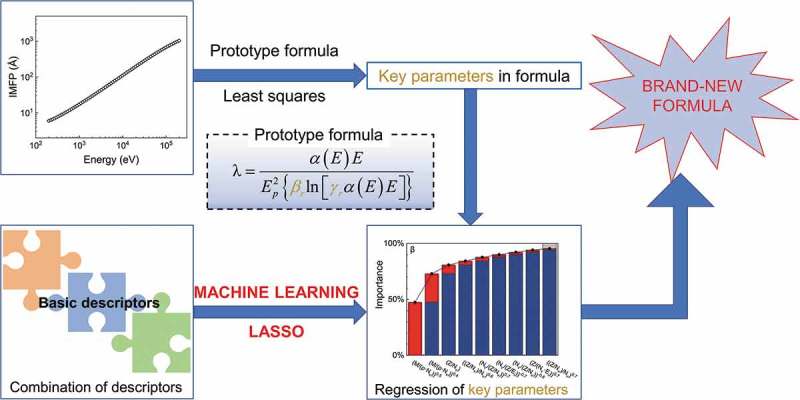

## 1. Introduction

The electron inelastic mean free path (IMFP) [1,2], which describes the mean distance an electron travels through a solid before losing energy, is of fundamental importance to electron-based surface analysis techniques, such as scanning electron microscopy, X-ray photoelectron spectroscopy, and Auger electron spectroscopy [2–7]. With a dielectric formalism, the IMFP can be calculated by various algorithms, such as Penn algorithm [8,9], Mermin algorithm [10–15] and ex-Mermin algorithm [16]. The full Penn algorithm (FPA) has been used to produce the largest IMFP database, and thus has had considerable influence in the field of surface analysis. In recent years, Tanuma et al. calculated IMFPs for 27 elemental materials [17,18], 15 inorganic compounds [19], and 14 organic compounds [20] in a wide energy range from 50 to 2000 eV. Furthermore, to increase the accuracy of IMFP calculations and expand the contents of the database, the IMFPs of 41 elemental materials [21] and 42 inorganic compounds [22] for energies up to 200 keV were calculated. We note that the database adopted here includes IMFPs for 41 elemental materials [21] and 42 compounds [22] calculated by the FPA.

Unfortunately, the calculations made by such algorithms and formulae need the energy loss function (ELF) [23] for the material of interest, which is usually difficult to obtain [24–27]. ELFs are, thus, still unavailable for many materials. To overcome this problem, researchers develop artificial empirical formulae whose independent variables are simple material-dependent parameters. In fact, researchers in the area of surface analysis tend to use empirical formulae instead of the FPA in application. Moreover, empirical formulae have a simple form that unifies the information of IMFP data. It is therefore of vital convenience for researchers to search for a relationship between IMFP data and material-dependent parameters. Although the use of empirical formulae may cause some accuracy loss, the formulae can be used quickly and have good descriptors for the definition of IMFPs.

As a starting point, the Bethe equation [28] for inelastic scattering was used in order to parameterize the IMFP data calculated or measured. All parameters of the equation are microscopic quantities. However, the original Bethe formula has an obvious shortcoming in that it is only valid for sufficiently high energies (above 200 eV).

Many formulae based on the Bethe formula (e.g. TPP-2M [21,22], G1 [29], and S1 [30]) have been derived successively. For example, Tanuma et al. [21,22] used macroscopic quantities for parameters in the Bethe formula while trying to extend the Bethe equation to low energies such as 50 eV. They established a new empirical formula, the TPP-2M formula. Two correction terms were introduced into the denominator to expand the energy range to lower energies. In addressing higher energies, a relativistic revision was made for the most recent version of the TPP-2M equation [21], allowing an accurate description of the IMFP. The use of the TPP-2M equation allows the convenient determination of the IMFP for a certain material and even the prediction of unknown IMFPs for some materials.

Although there are many formulae for predicting the IMFP, there are still problems to be solved, mainly relating to the artificial selection of the combination of terms. The combination space of terms is nearly infinite. The descriptions of several materials, such as carbon allotropes and boron nitride (BN), are very poor, because manually chosen terms can capture only relatively obvious physics of most materials, lacking both an overall and comprehensive understanding. Furthermore, Tanuma and co-workers have spent more than 20 years to build a database of IMFPs for elemental solids, inorganic and organic compounds, and to validate the applicability of the TPP-2M formula to many materials (see their initial work [17] to their most recent work [22]). Beyond the fitting work itself, however, one cannot ensure the applicability of the formula to materials not in the fitting database; that is, one cannot ensure generalization ability in machine learning (ML) terminology. Generally speaking, the manual selection of features is no longer efficient or even reliable.

In this work, we develop a framework instead of using the existing regression procedure, successfully avoiding the problems mentioned above. We first establish a suitable prototype formula and obtain values of key parameters using the prototype formula and the least-squares method. Meanwhile, a descriptor pool is established simultaneously using fundamental and important material-dependent parameters. We thus set the values of key parameters as a training target and descriptors in the pool as features. The Least Absolute Shrinkage and Selection Operator (LASSO) [31] is used to form the linear combination of the principle descriptors, which means the unimportant terms are automatically eliminated. Following this core step of ML, a brand-new empirical IMFP formula is produced, just after a process of merging similar terms and adjustments. Through this method, the new descriptors ensure robustness and generalization performance on all materials. Moreover, features selected from the data-driven degree are more likely to hold deeper physics meaning than features obtained in several attempts of using the TPP-2M formula, which is one of the most important aspects of our work. We note that this new framework is not limited to the formula for IMFPs but can be easily applied in other fields. The simplification of empirical formulae and the further discovery of information behind the terms in the formulae are superior aspects of our framework.

## 2. Methods

### 2.1. Lasso

LASSO [31] is a well-known set of techniques used in many data-driven statistical analyses in different fields. It provides low-dimensional solutions by recasting a problem into a convex minimization problem. That is to say, a sharp reduction in the number of terms (i.e. the number of descriptor selection characteristics) is mathematically achieved by solving a minimization function:

(1) $$\underset{w}{\arg\min}\frac{1}{2n}||Xw-y|{|}_{2}^{2}+\lambda ||w|{|}_{1},$$

where the first term is similar to a term in the least-squares algorithm while the second term is the so-called penalty term. This least-squares penalty term is combined with a constant *λ* and the *l1*-norm of the parameter vector ||*w*||_1_. On the one hand, a larger value of *λ* will eliminate more descriptors in the linear regression; on the other hand, the use of the *l1*-norm ||*w*||_1_ is crucial. In fact, the shrinkage function of LASSO relies on this *l1*-norm.

**Figure 1.**
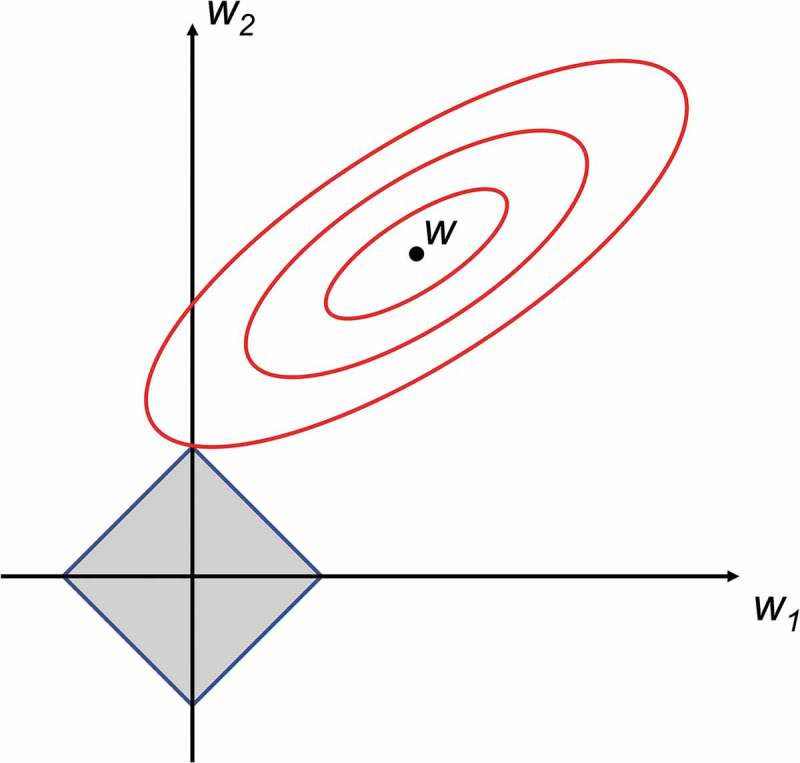
A simple LASSO algorithm application example of two-dimensional descriptors. Here w_1_ and w_2_ are the two dimensions of the target parameter. The red ellipse is the branch of values for target parameter vector *w* and the square below represents the *l1*-norm in LASSO.

Figure 1 is a simple LASSO algorithm application example of two-dimensional descriptors. The red ellipse is the branch of values for target parameter vector *w*. The value of ||*w||* on the same ellipse is the same, and a smaller ellipse corresponds to a better value of *w*. The square centered on the origin in Figure 1 represents the set of points that satisfy the constraints of the *l1*-norm in Equation (1); only points that fall into the square can be selected. An optimization method is applied, and the estimated value of LASSO is the intersection of the ellipse and the square below. Unless the ellipse is exactly tangential to the square on one side of the rectangle, the intersection will fall on the vertices of the rectangle, and the estimated value of a parameter will be compressed to zero. That is to say, the variable has been removed from the model. If the penalty term uses not the *l1*-norm but say the *l2*-norm, the square in Figure 1 is a circle. The ellipse will have a low possibility of intersecting with the vertices of the *l2*-norm circle, and there is no shrinkage ability in this case. This penalty term is the stakeholder in LASSO.

### 2.2. Cross validation

Our new formula fits the IMFP with high accuracy similar to or even better than that of the TPP-2M formula owing to the chosen descriptors. Such accuracy is achieved for all materials in the dataset because a traditional *k*-fold cross validation (CV) [32] is naturally used in our ML work. In *k*-fold CV, the original sample is randomly partitioned into *k* equally sized subsamples. Of the *k* subsamples, a single subsample is retained as the validation data for testing the model, and the remaining *k* − 1 subsamples are used as training data. The CV process is then repeated *k* times, with each of the *k* subsamples used exactly once as the validation data. The *k* results can then be averaged to produce a single estimation.

We note that LASSO and CV used in this work are powered by the Scikit-learn library [32].

### 2.3. Details of building the descriptor pool

Our goal is to create combined descriptors that have physics meanings. Generally speaking, the completeness of descriptors that hold the same complexity must be ensured in this establishment procedure, but without introducing unphysical operations or quantities. Here a step-by-step framework like that shown in Table 1 is applied to the combination procedure.

**Table 1.** Feature combination framework based on seven basic features.

ID	Description	#
A1	7 basic features	7
B1	$\left(E_{i}+E_{g}\right);\left(E_{i}-E_{g}\right);\left(E_{i}^{2}+E_{g}^{2}\right);\left(E_{i}^{2}-E_{g}^{2}\right)$	61
B2	f∙g; f,g∈{A1}	
B3	f/g; f,g∈{A1}	
C1	f∙g; f∈{A}, g∈{B}	1136
C2	f/g; f,g∈{A,B}	
D1	f^i^, f∈{A,B,C}, i∈{-0.9, …, -0.1,0.1, …, 0.9}	16,524

- The starting point is the seven basic features shown as series A in Table 1. The introduction and necessity analysis has been discussed in results and discussion part.
- The first step is vital in establishing a well-formed combination of descriptors. This is because the descriptors created in each step strictly relate to those created in the last step. The first combination is shown in Table 1 as series B (including B1, B2, and B3). The decision point in series B considers the physics meanings of descriptors; therefore, summation and difference operations between inhomogeneous quantities, such as *E*_i_ + *Z* and *E*_i_^2^ + *E*_g_, are not accepted. A serious observation of the seven basic features reveals that only energies (*E*_g_ and *E*_i_) can be combined like series B1 (limited to quadratic terms). Moreover, a multiplication or division operation will not create inhomogeneous quantities, like descriptors in series B2 or B3 in Table 1. We note that owing to zero values existing for some basic features, descriptors with divided-by-0 problems are automatically excluded.
- On the basis of series A and B, more complex descriptors can be combined. In series C (including C1 and C2), descriptors in series B are further multiplied or divided or divided by basic features in series A. This procedure raises the complexity of the descriptor by one step and ensures that all descriptors of the same complexity are included. Although there will obviously be repeated descriptors, as seen in the result, we use a linear regression after LASSO to merge similar terms. This type of step-by-step procedure can theoretically be repeated time after time, but this is not done here considering the calculation ability of the program and for the sake of simplicity; further analysis can be seen in the results and discussion sections.
- Referring to the original terms in TPP-2M [21,22] and other formulae [29,30] previously developed and the need for a root operation, powered terms are used for series A, B, and C to make series D. This series has the largest volume in the descriptor pool and provides alternative choices for precise terms in the formula.

Descriptors in the pool are created as described above. All descriptors in series A, B, C, and D are used with LASSO. This automatic brute-force and step-by-step method of establishing a descriptor pool can be used in normal empirical-formula regression work. The framework can enumerate the descriptors needed, fulfilling the need of corresponding complexity, and thus has good control of the detailed operation base for specific needs of the empirical formula.

## 3. Results and discussion

### 3.1. Selection of the prototype formula

The prototype formula and target values of the key parameters in the formula must first be decided. The first point is the prototype formula. Early work by Bethe [28] treated inelastic scattering by atoms and established the so-called Bethe theory and Bethe formula for the description of energy dependence of inelastic cross sections.

Tanuma et al., then, proposed the following a predictive equation for IMFP over 200 eV based on the Bethe formula [33].

(2) $$\lambda =\;\frac{E}{E_{p}^{2}\left[\beta \;\ln\left(\gamma E\right)\right]},$$

where λ is the IMFP, *E* is the electron energy, *E*_p_ is free-electron plasmon energy, and *β* and *γ* are parameters. They determined general formula for these parameters based on the IMFP data over 200 eV to 2000 eV for only 31 materials.

Meanwhile, the most compatible and well-received mature formula is the relativistic TPP-2M formula [21,22]. It has a wider applicable energy region (50 eV – 200 keV) but its performance at energies lower than 100 eV is not reliable. This must be due to the limitations of the accuracy of the used IMFP database at low energies.

**Figure 2.**
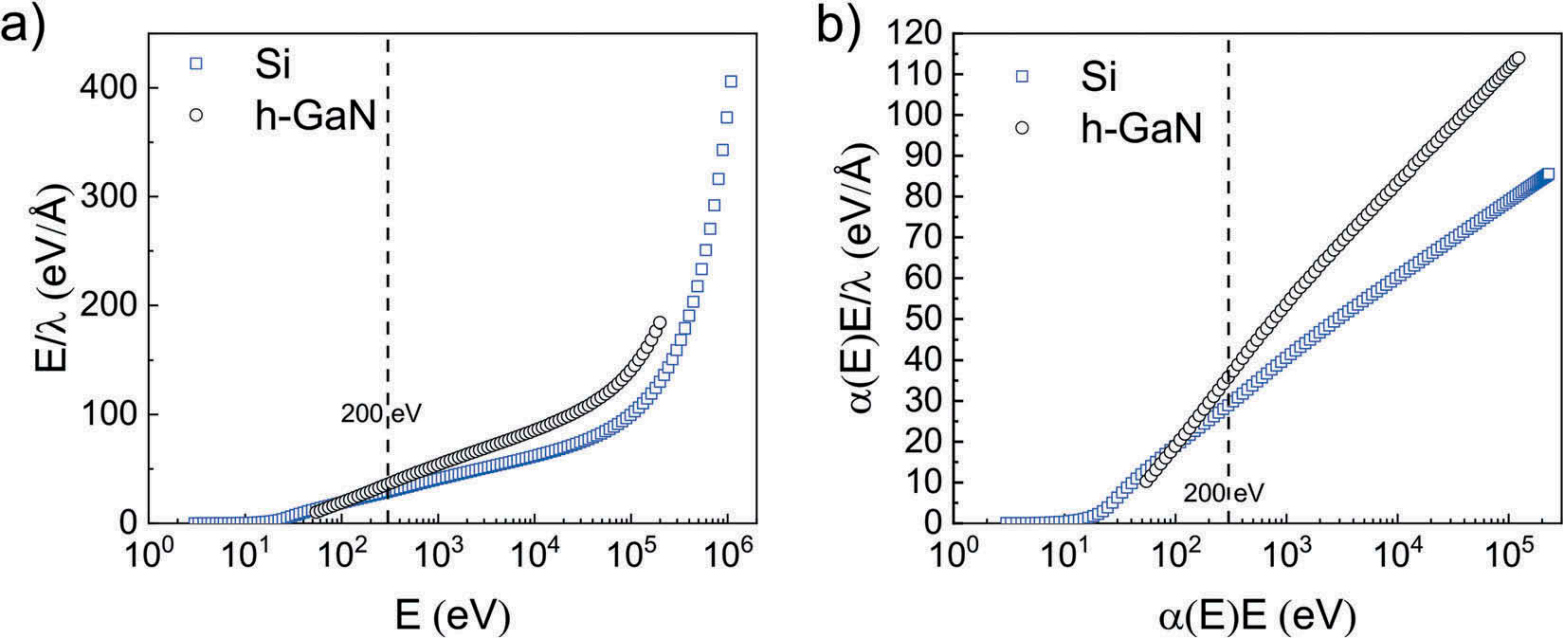
(a) Non-relativistic and (b) relativistic Fano plots for Si (an exemplary representative of elemental materials, open circles) and h-GaN (an exemplary representative of compounds, open squares).Two energy ranges are considered: from 50 eV to 1 MeV for elemental materials and from 200 eV to 200 keV for compounds.

The prototype can be treated as a modified Bethe formula:

(3) $$\lambda =\frac{\alpha \left(E\right)E}{E_{p}^{2}\left\{\beta_{r}ln\left[\gamma_{r}\alpha \left(E\right)E\right]-\frac{C_{r}}{E}+\frac{D_{r}}{E^{2}}\right\}},$$

where *α*(*E*) is the relativistic modification term only associated with E while *β*_r_, *γ*_r_, *C*_r_, and *D*_r_ are parameters to be determined at each target material. It is seen that the modified Bethe formula has *C*_r_ and *D*_r_ for one and two more order corrections compared with the original Bethe formula. *α*(*E*) is then introduced to meet the requirement of higher energy (>10 keV). The necessity of the modification terms can be validated by using a Fano plot in which (*E*/*λ*) is plotted versus ln*E*. The need for the additional terms can be seen from the Fano plots if the data points lie sufficiently close to a straight line in Figure 2(a).

Figure 2(a) shows a clear linear relationship at energies ~200 eV and has a uniform rising trend at energies higher than 10 keV. The values of C and D are effective only at energies lower than 200 eV as was shown in Equation (2) [33].

For the higher energy region, however, Shinotsuka et al. [21] reported that the trend is due to the relativistic effect, which is not negligible in the higher energy region, showing that the *α*(*E*) term is necessary. Then, we present a relativistic Fano plot (Figure 2(b)) in which (*α*(*E*)*E*/*λ*) versus ln*α*(*E*)*E* has a good linear relationship in our selected energy region above 200 eV. We have

(4) $$\lambda =\frac{\alpha \left(E\right)E}{E_{p}^{2}\left\{\beta_{r}ln\left[\gamma_{r}\alpha \left(E\right)E\right]\right\}},$$

which we refer to as the TPP-LASSO formula. The *E*_p_ and some of the basic features that are mentioned later are extracted from the literature Refs [21,22]. We have also made some attempts showing that Equation (4) is suitable for this work as the prototype formula.

### 3.2. Fitting of the parameters in the prototype formula

Another consideration is the values of key parameters, namely $\beta_{r}$ and $\gamma_{r}$. The least-squares method can be applied to a selected IMFP database and TPP-LASSO formula to fit $\beta_{r}$ and $\gamma_{r}$. The essential problem is thus to select a robust IMFP database. Fortunately, through decades of study, a large quantity of IMFP results has been accumulated to serve as a reliable database with which to build the ML model. Shinotsuka et al. [21] theoretically computed the IMFP with the FPA for 41 elemental materials that have complete data of optical constants over a wide energy range. Shinotsuka et al. [22] similarly calculated IMFPs for 42 compound materials. These IMFP data for 83 materials can be included in the initial database for the fitting of key parameters. However, the low-energy (<50 eV) IMFPs calculated with FPA are not reliable. We thus adopt only IMFPs above 200 eV. The information for the 83 solids is therefore included in the model to obtain the parameters.

According to the target formula, *β* and *γ* are fitted using the least-squares method. Here, the accuracy of the fitting is measured as the root-mean-square deviation (RMSD):

(5) $$RMSD=\sqrt{\frac{1}{n}\overset{n}{\underset{i=1}{\sum }}{\left(\frac{\lambda_{fit}\left(E_{i}\right)-\lambda \left(E_{i}\right)}{\lambda \left(E_{i}\right)}\right)}^{2}},$$

where *n* is the total number of data points in the dataset, $E_{i}$ is the electron energy, $\lambda_{fit}\left(E_{i}\right)$ is the IMFP calculated using our fitted *β* and *γ*, and $\lambda \left(E_{i}\right)$ is the target value calculated by the FPA. The predication improves as the RMSD approaches zero. The fitting quality can be measured through the RMSD stated here. The RMSD averaged across all materials is 1.8%, with the deviation being a maximum for Ni (3.0%). The fitting of *β* and *γ* is relatively accurate and can be applied to the LASSO procedure as training data.

### 3.3. Establishment of the descriptor pool

Ghiringhelli et al. recently introduced ML and data-driven concepts to material science [34]. In their work, they gave a framework for the choice of the set of descriptive parameters (termed descriptor) to reveal the scientific connection between the descriptor and the actuating mechanisms. They not only provided the descriptor piling-up framework but also suggested LASSO as the latest ML method [34] to select important descriptors. We believe that this method will be vital to work on formula regression, which is relevant to the present work. Utilizing the ‘feature selection’ characteristic, we can search for descriptors from a large quantity of descriptors that we enumerated to describe the IMFP better than ever. The next part in building the database for ML is thus to select proper input parameters, namely the descriptor of the material feature. Following ‘feature selection’ for finding the best descriptors in Ref [34]., it is apparent that a complex build of the feature space is required.

According to the flow presented in Table 1, the starting point is the seven basic features (series A). The features are *Z* (atomic number), *M* (atomic mass), *ρ* (density), *N*_v_ (number of valence electrons per atom), *E*_g_ (bandgap energy), *E*_i_ (starting-point energy), and *R* (atomic radius). First, it is obvious that some of the features are for elemental materials and not compatible to compounds. Our solution is to extend their definitions to the compounds such that they are reasonable. For *Z*, the total number of electrons per molecule is used for compounds; for *M*, the molecular mass is used instead; for *R*, the molecular average of the radius for all atoms per molecule is used instead, similar to the case for element materials. Second, *E*_i_ is a new feature and is valence-band width plus the band gap energy. That is to say, the starting point of electron energy in the TPP(−2M) formula is the Fermi energy (*E*_F_) for conductors [21] and bottom of the conduction band for non-conductors [22]. *E*_i_ is defined as such for element materials and compounds because we believe that the starting point of the electron energy has its physical and distinguished meaning in the mix of element and compound materials. On the basis of the most basic features, a step-by-step combination is carried out to establish the descriptor pool (series B, C, and D), ensuring the completion of descriptors in each step but excluding inhomogeneous descriptors. Detailed information can be found in the Methods section and Table 1.

### 3.4. Selecting principle terms with LASSO

LASSO is run on the set of ~17,000 candidate descriptors. The least-squares results for *β* and *γ* were set as the target values; that is, *y* values in Equation (1) of the LASSO method. Cross-validation (CV) is adopted naturally to adjust the hyper-parameters in LASSO. With the best hyper-parameters obtained by CV, 23 descriptors for *β* and 29 descriptors for *γ*, whose coefficients are not zero, are selected. Coefficients of the other descriptors are reduced to zero on the basis of the ‘feature selection’ of LASSO. The TPP-LASSO formula is built with our framework, while *β* and *γ* are the linear combination of the descriptors together with the intercept, and coefficients are also given by LASSO.

Despite the quantity of descriptors sharply reducing from ~17,000 to ~30, only 0.2% principle descriptors are selected, there are still too many descriptors (terms) for a formula. A natural proposal is to select descriptors according to their importance, but it is difficult to see the importance directly. Here an importance measurement is introduced intuitively. The importance of the *m*-th descriptor for a certain material can be measured as

(6) $$I_{m}=\frac{\left|a_{m}F_{m}\right|}{\sum_{i=1}^{n}\left|a_{i}F_{i}\right|},$$

where *a* is the coefficient of descriptors, *F* is the descriptor value, and *n* is the total number of descriptors. A larger value of *I*_m_ corresponds to a more important descriptor. The sum of *I*_m_ for all descriptors should be 100% for a certain material. To obtain the overall importance of the *m*-th feature for all materials, the average importance of *β* and *γ* for all materials is calculated.

**Figure 3.**
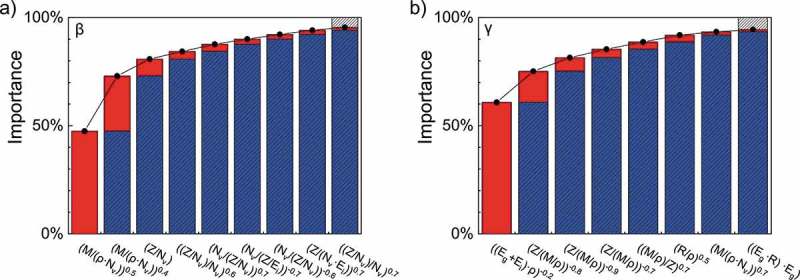
Percentage histogram of the importance ratio for LASSO-selected descriptors for a) *β* and b) *γ*. Only descriptors with an importance ratio greater than 1% are shown in detail. The upper shadowed parts in the columns on the right of each panel summarize the importance of descriptors with minor importance (<1%). The upper red parts represent the importance increase when terms are accumulated following the importance order from high to low. We note that the importance of the constant term, namely, the intercept of the linear combination, is included in the importance of the first term.

Figure 3 shows the accumulation process when descriptors are added in turn. For *β*, (*M*/*ρN*_v_)^0.5^ and (*M/ρN*_v_)^0.4^ obviously have the largest proportions except for the intercept and are considered the main descriptors, while *Z/N*_v_ and others are considered the correction. Similarly, for *γ*, [(*E*_g_ + *E*_i_)*ρ*]^−0.2^ is the main descriptor and (*Zρ/M*)^−0.8^ and others are the correction. It naturally follows from this train of thought to shrink the quantity of descriptors and omit the unimportant terms in the formula. Therefore, only three descriptors of *β* and two descriptors of *γ* are used for our shortened TPP-LASSO formula, which we refer to as the TPP-LASSO-S formula. Considering that the coefficients of the descriptors for the shortened formula may no longer be precise, the linear regressor is used to update the formula for the same IMFP database. Our TPP-LASSO-S formula is given by

(7a) $$\lambda =\frac{\alpha \left(E\right)E}{E_{p}^{2}\left\{\beta_{r}ln\left[\gamma_{r}\alpha \left(E\right)E\right]\right\}}\left(\text{\textbackslash{}AA}\right),$$

(7b) $$\beta_{r}=-0.0012+0.046{\left(\frac{M}{\rho N_{v}}\right)}^{0.5}-0.035{\left(\frac{M}{\rho N_{v}}\right)}^{0.4}\text{\textbackslash{}break}\;\;\;\;\;\;\;\;\;\;\;\;\;\;\;\;\;\;\;\;\;\;\;\;\;\;\;\;\;\;\;\;\;\;\;\;\;\;\;\;\;\;\;\;\;\;\;\;\;\;\;\;\;\;\;\;\;\;\;\;\;\;\;+0.0019\frac{Z}{N_{v}},$$

(7c) $$\gamma_{r}=-0.07+0.26[\;\rho (E_{i}+E_{g}){]}^{-0.2}+0.066{\left(\frac{Z\rho }{M}\right)}^{-0.8}.$$

where $\alpha \left(E\right)={\left[1+\left(E/2m_{e}c^{2}\right)\right]/\left[1+\left(E/m_{e}c^{2}\right)\right]}^{2}$, and $m_{e}c^{2}$ is the electron rest energy (510,998.9eV), *E*_p_ is the free-electron plasmon energy (in eV), *E*_i_ is the starting-point energy (in eV), *E*_g_ is the bandgap energy for nonconductors (in eV), *ρ* is the bulk density (in g cm^−3^) and *N*_v_ is the number of valence electrons per atom or molecule.

### 3.5. Necessity of ~17,000 candidate descriptors

Table 1 shows that the descriptor pool has been established in our framework has for the major parts step-by-step, namely A, B, C, and D, a total of ~17,000 descriptors. This quantity of descriptors is appropriate while considering together accuracy, stability, formula length, and calculation consumption. To allow discussion of this statement, a series of different simplified TPP-LASSO formulae were produced with repeats of the entire framework, in which the procedures were the same except for the descriptor pool size. The simplification for each TPP-LASSO formula was conducted using the least-squares method and the target term quantities of *β* and *γ* were the same as or less than that in Equation (7) to allow fair comparison.

**Figure 4.**
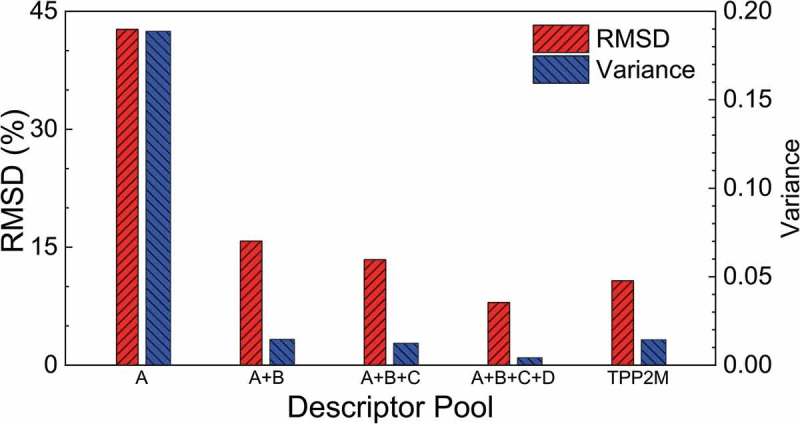
Comparison of average percentage RMSDs and variation of RMSDs for different TPP-LASSO-S formulae produced using our framework with different sizes of descriptor pool. The x-axis represents the size of the descriptor pool. The red columns indicate average RMSDs and blue columns show the variation of RMSDs. The TPP-2M formula is included in the last group of columns to allow direct comparison.

Figure 4 compares the average RMSDs and variation of RMSDs for the different simplified TPP-LASSO formulae produced. On one hand, the simplified TPP-LASSO formulae produced using less than the A + B + C + D descriptor pool showed poorer accuracy and stability compared to those obtained using the original TPP-2M formula. This represents the shortage of quantities if the descriptor pool does not reach step D. On the other hand, the A + B + C + D descriptor pool that included all the descriptors displayed the same complexity as the terms in the TPP-2M formula but achieved better performance. The above discussion reveals that using the A + B + C + D descriptor pool in our framework is quite accurate and stable and does not introduce unnecessary complexity.

In addition, we also validated the robustness of LASSO by using different sizes of training dataset randomly extracted from the set A + B + C + D. It turns out that LASSO can pick out the five principle descriptors in *β* and *γ* appearing in our simplified TPP-LASSO formula, when given reasonable different contents and sizes of training sets. This also reflects the steadiness of our principle descriptors pick up by LASSO.

### 3.6. Comparison of our new formula and other formulae

As previously mentioned, many empirical formulae stand parallel within the field of describing IMFPs, especially formulae applicable in similar energy ranges. Formulae for high-energy electrons are now considered for comparison.

Gries proposed the so-called G1 formula [29] using an atomistic model:

(8) $$\lambda =\frac{k_{1}V_{a}E}{Z^{*}(\log E-k_{2})}\left(nm\right),$$

where $V_{a}=M/\rho$ is the atomic volume, Z* is the nominal effective number of interaction-prone electrons per atom, which was found to equal *Z*^0.5^, and average values per atom of *M* and Z* are used for compounds. $k_{1}$ and $k_{2}$ are fitting parameters; Tanuma et al. [35] summarized their best values on the basis of Gries’ work. The most inconvenient point is that $k_{1}$ and $k_{2}$ values are given separately for each group of material relating to the periodic table. This so-called G1 formula, has better performance for several compounds but there can be substantial deviations (approximately 50%) for some materials.

Another empirical expression, designated the S1 formula, was proposed by Seah [30] to estimate IMFP values for materials:

(9a) $$\lambda =\frac{\left(4+0.44Z^{0.5}+0.104E^{0.872}\right)a^{1.7}}{Z^{0.3}\left(1-W\right)}\left(nm\right),$$

(9b) $$a^{3}=\frac{10^{21}M}{\rho N_{A}\left(g+h\right)},$$

where *W* = 0.02*E*_g_, (*W* = 0 for an elemental solid) and *N*_A_ is the Avogadro constant. The terms *g* and *h* in Equation (9b) represent stoichiometry coefficients for assumed binary compound G_g_H_h_; for an elemental material, *g* = 1 and *h* = 0. The S1 formula is not following the consideration of Bethe equation thus loses some of the physics image. As a result, the S1 formula is relatively accurate for most elemental materials but the adjustment for compounds is clearly insufficient, leading to a poor description for them. Furthermore, the S1 formula cannot be further expanded to a multiple compound like Y_3_Al_5_O_12_.

**Figure 5.**
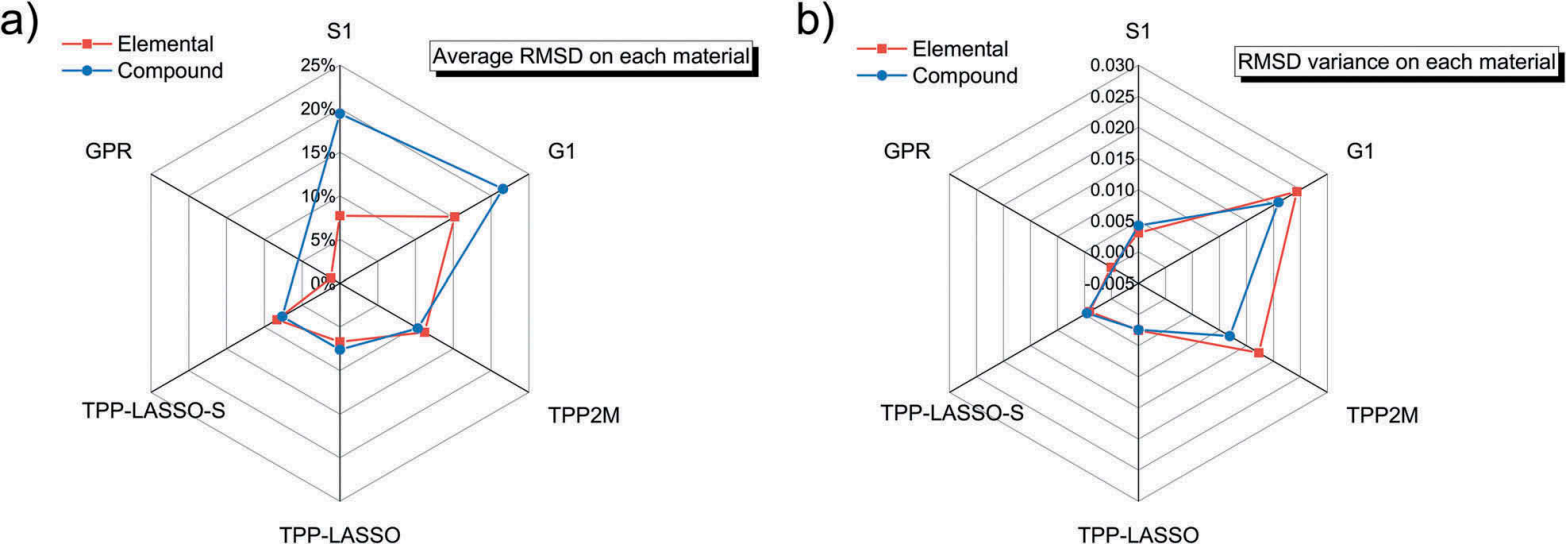
Comparison of (a) average percentage RMSDs and (b) variation in RMSDs of for all 83 materials when using the S1, G1, TPP-2M, TPP-LASSO, and TPP-LASSO-S formulae and a machine learning method (i.e. the GPR). The red line is for elemental materials and the blue line for compounds.

Figure 5 compares our TPP-LASSO formulae and other empirical formulae mentioned above, showing the RMSD and variance. S1, G1, and TPP-2M formulae are not optimized for the newly calculated IMFP database, namely the FPA results. Therefore, some of the formulae may not applicable to some materials, for which these materials will be neglected in the statistics of corresponding formula. We multiplied the electron energy by *α*(*E*) in the comparison because the S1 and G1 formulae do not considering relativistic modification in the high energy region.

In the degree of horizontal comparison, Figure 5(a) focuses on the accuracy of the formulae. The RMSDs of the new formulae are lower than 10%, while other formulae cannot achieve such accuracy for both elemental materials and compounds, even if unsuitable materials are ignored. Beyond looking at accuracy, Figure 5(b) shows the RMSD variance for each formula. The figure reflects the stability of the IMFP description, or the generalization ability in terms of ML. To put it simply, there are barely any obviously poorly described materials owing to the contribution of CV, and the variances are lower than 0.005 for our formulae.

In contrast, there are many extremely high-RMSD materials for some other formulae. For example, the RMSD of diamond is as high as 71% according to the TPP-2M formula. In another degree of vertical comparison, S1 and G1 formulae provide relatively accurate and stable descriptions of elemental materials but poor descriptions of compounds; the TPP-2M formula has the same level of description accuracy for elemental materials and compounds, while it has poor stability because of outliers like the carbon allotropes mentioned above. So far, our formulae are seen to be not only accurate but also stable and all-round.

To make a uniform comparison, a recently proposed ML method, namely the Gaussian process regressor (GPR) [36], was used to predict the IMFP for elemental material (details of the prediction of IMFP using GPR will be presented elsewhere). It is seen that the accuracy of our TPP-LASSO formula is between that of the GPR and the accuracies of other formulae; however, our TPP-LASSO formula and the GPR have similar stabilities. This reveals the advantage of our new formula over other empirical formulae due to the introduction of the ML element. It is noted that the formula proposed by Nguyen-Truong [37] has a decisive weakness in that it does not apply simple material parameters and it is thus extremely reliant on the ELF. Additionally, his formula is derived from a (infinitive) high-energy approximation of the FPA, resulting in this formula being inapplicable at energies below 500 eV. Although his formula has a powerful fitting performance in the high energy region, such an analytical formula is not appropriate for comparison here.

**Figure 6.**
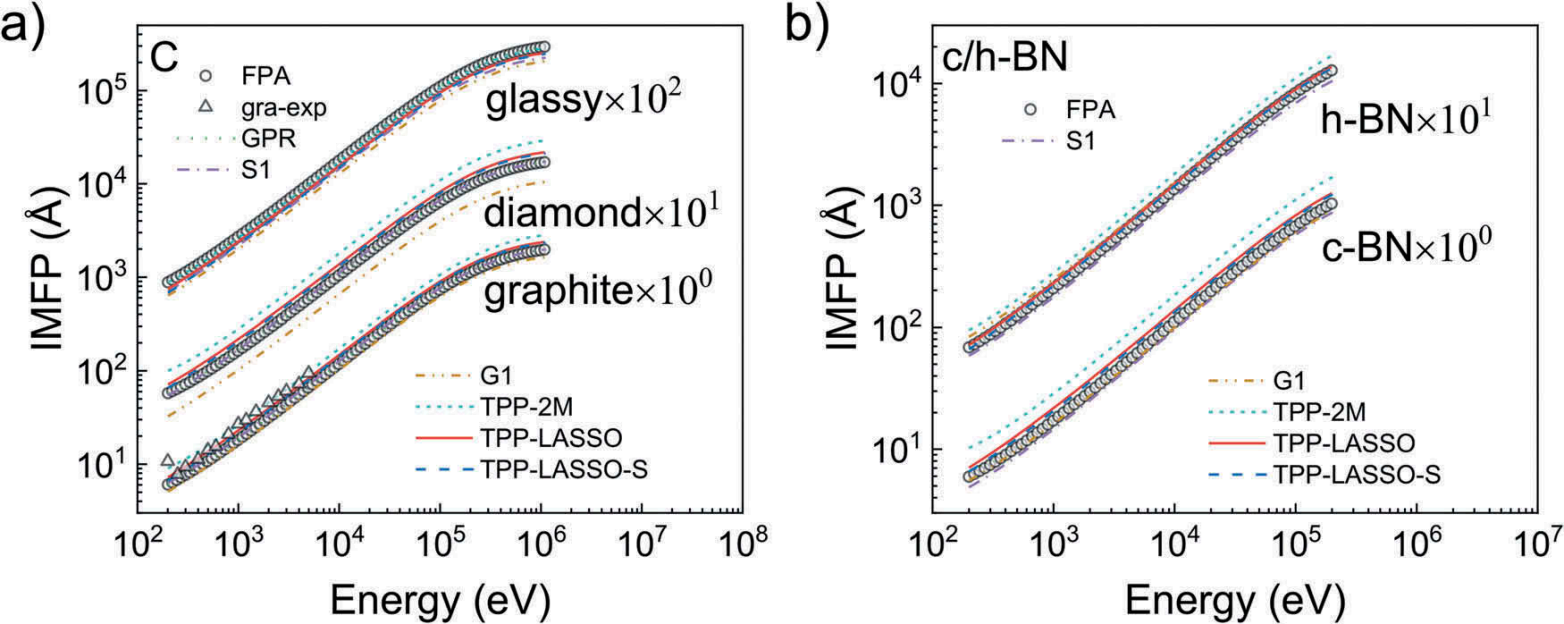
Comparison between the FPA-calculated IMFP values (black hollowed dots), the experimental IMFP values for graphite carbon (black hollowed triangles), the IMFP described by GPR (green dot line), S1 formula (purple dash-dot line), G1 formula (brown dash-dot-dot line), TPP-2M formula (indigo short-dash line), TPP-LASSO formula (red solid line), and TPP-LASSO-S formula (blue dash line). The results of three typical carbon allotropes are shown in a) while the results of c/h-BN are shown in b).

For most materials, our formulae better describe the IMFPs for most materials than those of TPP-2M formula; i.e. our formulae have lower average RMSDs than the other empirical formulae considered. Table 2 compares the RMSDs in detail. Numerically speaking, average RMSDs on all materials are 7.2% and 8.0% for the non-simplified and TPP-LASSO-S formulae and 10.8% for the TPP-2M formula, showing an improvement of nearly one-third. In fact, 50 out of the 83 materials in the case of the TPP-LASSO formula and 52 out of the 83 materials in the case of the TPP-LASSO-S formula have accuracies better than those when using the original TPP-2M formula. Furthermore, materials poorly described by the TPP-2M formula, such as the three carbons and two types of Born Nitride as shown in Table 2, are accurately described by our new formula. Detailed comparisons are presented in Figure 6. Our TPP-LASSO formula poorly describes the five materials in the figure but does a better job than the TPP-2M formula. We note that carbon allotropes have similar RMSDs according to our TPP-LASSO-S formula.

**Table 2.** Comparison of percentage RMSDs calculating from Equation (5) among TPP-2M, TPP-LASSO, and TPP-LASSO-S formulae.

RMSD elemental	TPP-2M	TPP-LASSO	TPP-LASSO-S	RMSD compounds	TPP-2M	TPP-LASSO	TPP-LASSO-S
Li	16.1%	7.5%	8.8%	AgBr	9.4%	6.5%	7.0%
Be	21.0%	2.7%	19.5%	AgCl	8.0%	7.0%	6.4%
C-graphite	45.2%	5.0%	18.9%	h-AgI	9.0%	5.5%	8.1%
C-diamond	71.2%	5.1%	24.8%	Al_2_O_3_	18.1%	3.4%	4.0%
C-glassy	2.1%	24.2%	16.4%	AlAs	0.8%	3.9%	2.2%
Na	3.8%	6.3%	6.1%	h-AlN	13.9%	2.2%	4.1%
Mg	8.8%	9.3%	13.5%	AlSb	3.9%	8.0%	2.9%
Al	8.7%	4.7%	12.6%	c-BN	66.0%	10.8%	19.2%
Si	4.1%	6.1%	2.2%	h-BN	33.3%	2.1%	4.5%
K	2.4%	0.6%	4.9%	h-CdS	10.4%	9.4%	9.2%
Sc	25.4%	20.6%	26.1%	h-CdSe	12.2%	9.5%	10.1%
Ti	19.7%	9.3%	19.6%	CdTe	7.5%	3.2%	5.7%
V	7.6%	4.0%	8.9%	GaAs	4.1%	7.0%	3.3%
Cr	3.8%	5.8%	6.2%	h-GaN	3.0%	3.4%	6.0%
Fe	4.0%	9.9%	2.2%	GaP	3.0%	6.3%	4.4%
Co	4.6%	4.0%	12.4%	GaSb	9.1%	11.3%	4.5%
Ni	3.1%	3.9%	7.9%	h-GaSe	0.9%	6.1%	1.9%
Cu	8.9%	10.7%	4.3%	InAs	9.2%	9.0%	3.4%
Ge	3.5%	2.1%	4.5%	InP	6.8%	9.7%	5.3%
Y	13.3%	1.4%	3.8%	InSb	14.0%	13.2%	4.6%
Nb	1.8%	14.6%	7.4%	KBr	5.7%	11.7%	20.3%
Mo	5.0%	5.3%	3.2%	KCl	4.5%	11.7%	18.7%
Ru	2.9%	1.9%	7.5%	MgF_2_	20.9%	9.0%	13.5%
Rh	5.0%	3.8%	11.9%	MgO	10.0%	7.7%	5.4%
Pd	2.8%	2.1%	10.0%	NaCl	16.5%	27.5%	32.1%
Ag	3.1%	5.3%	10.8%	NbC_0.712_	2.1%	4.5%	0.9%
In	20.5%	4.1%	2.8%	NbC_0.844_	2.4%	4.5%	1.0%
Sn	1.7%	13.4%	10.8%	NbC_0.93_	2.6%	4.6%	1.1%
Cs	32.3%	4.3%	2.8%	PbS	6.2%	3.4%	1.2%
Gd	7.6%	16.1%	10.7%	PbSe	9.0%	3.8%	2.1%
Tb	7.7%	1.8%	4.1%	PbTe	15.4%	7.7%	1.7%
Dy	2.7%	8.4%	3.1%	SiC	15.1%	2.6%	8.3%
Hf	12.6%	10.7%	9.1%	SiO2	2.8%	19.4%	22.9%
Ta	15.0%	5.2%	2.3%	SnTe	11.9%	15.9%	7.1%
W	6.8%	5.5%	2.4%	TiC_0.7_	14.0%	4.9%	13.1%
Re	4.4%	2.7%	3.2%	TiC_0.95_	17.1%	6.9%	15.4%
Os	7.8%	7.8%	4.7%	VC_0.76_	3.4%	4.3%	3.8%
Ir	8.2%	4.4%	3.1%	VC_0.86_	5.2%	2.7%	5.5%
Pt	10.9%	4.8%	3.3%	Y_3_Al_5_O_12_	1.4%	5.6%	5.1%
Au	10.8%	5.2%	3.5%	ZnS	4.9%	10.4%	9.5%
Bi	12.9%	4.5%	1.8%	ZnSe	11.4%	9.5%	9.6%
				ZnTe	8.3%	3.4%	5.3%

Besides the comparison of IMFPs between those of different formulae and those of FPA result, here we also compare IMFPs by TPP-2M formula and those by our TPP-LASSO-S formula with the experimental IMFPs from Tanuma et al. [38]. The comparison for graphite for electron energies above 200 eV is shown in Figure 6(a). The experimental result is closer to the IMFPs of our TPP-LASSO-S formula than those of TPP-2M formula except the data point at 200eV. We also show the comparison results for electron energies above 200 eV as RMSD (Equation (5)) for different materials as below (before the slash is the RMSD between TPP-2M formula and experimental IMFPs; after the slash is the RMSD between our TPP-LASSO-S formula and experimental IMFPs): graphite carbon (14.0%/15.2%), Si (11.2%/9.4%), Cr (14.3%/15.5%), Fe (9.3%/12.3%), Cu (5.2%/8.2%), Mo (18.8%/16.1%), Ag (11.26%/15.5%), Ta (29.3%/11.0%), W (29.7%/21.6%), Pt (11.7%/16.7%), Au (7.6%/8.7%), Average (14.8%/13.7%). The accuracy of our TPP-LASSO-S formula is slightly superior to the TPP-2M formula. The RMSDs of Ta and W, however, are greatly decreased through our formula. This must be a clear evidence that our formula could increase the accuracy of poorly descripted materials in TPP-2M formula, without any large accuracy sacrifice of other materials.

### 3.7. Physics picture behind the principle terms

Information is buried deep in Figures 5 and 6. First, it is possible that our TPP-LASSO formula outperforms the TPP-2M formula because it has many more terms. However, the RMSD difference between the TPP-LASSO formula and TPP-2M formula and the difference between TPP-LASSO and TPP-LASSO-S formulae reveals that the number of terms is not an important factor; i.e. the TPP-LASSO-S formula does not lose its advantage when the number of descriptors is similar to that for the TPP-2M formula. Second, it is noted that if there is a large RMSD fluctuation among different materials, the generalization capability of the selected descriptors is probably poor. The extremely large RMSDs of the five typical materials for the original TPP-2M formula are such examples of poor generalization capability, which is considered the greatest weakness of the TPP-2M formula. For our TPP-LASSO formula, the generalization capability is so good that irrespective of where the number of descriptors is limited, the IMFP is described with relatively uniform accuracy. The similar RMSDs for our TPP-LASSO-S formula among carbon allotropes demonstrate the generalization capability in different cases.

On the basis of the reliability of our new formula and the descriptors selected, we finally turn to the physics meaning behind the descriptors that we found. Figure 3 shows that the most important descriptors are (*M/ρN*_v_)^0.5^ and (*M/ρN*_v_)^0.4^. Surprisingly, the definition of *E*_p_ is

(10) $$E_{p}=28.8{\left(\frac{\rho N_{v}}{M}\right)}^{0.5}\left(eV\right).$$

In other words, the main descriptors we found for *β* are actually ${E_{p}}^{-1}$ and ${E_{p}}^{-0.8}$. So far, one of the most effective descriptors found manually to describe the IMFP is *E*_p_. *E*_p_ has been used in the TPP-2M formula following the initial work of Tanuma et al. [17], for valence electrons make the main contribution to electron scattering in a bulk and *E*_p_ contain *N*_v_ in the formulae. We believe that this interesting fact is not just a coincidence and that there is a physics meaning behind it. As expected, once Tanuma et al. have visited to explain the magnitude of the IMFP on element materials in Ref [39]. Tanuma et al. compared the theoretical calculated IMFP formula and TPP-2M formula and concluded that

(11) $$\beta ≅\frac{k}{E_{a}},$$

where *k* is a constant and $\Delta E_{a}$ is the average excitation energy. In the discussion in Ref [39]., a hypothesis is to use *E*_p_ as the candidate of $\Delta E_{a}$, and it is thus concluded that *β* ~ 1/*E*_p_.

**Figure 7.**
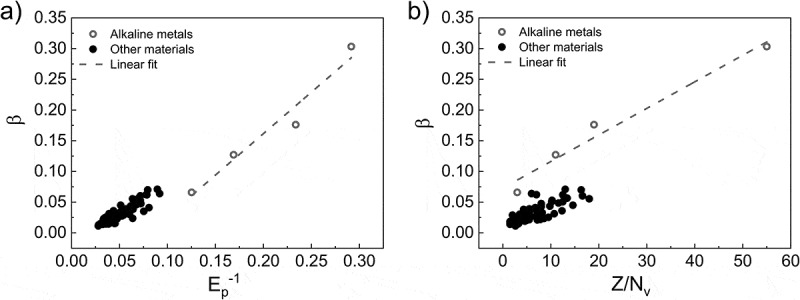
Relationships of the principle descriptors with *β*: (a) *E*_p_^−1^ and (b) *Z*/*N*_v_. Open circles represent the deviating data for alkaline metals and the corresponding linear fit.

In our work, however, 1/*E*_p_ is selected as the most important descriptor out of ~17,000 descriptors. This is evidence that in a very large space, 1/*E*_p_ is the most suitable descriptor of the IMFP. In Figure 7(a), *β* has a clear linear relationship with ${E_{p}}^{-1}$, similarly to that with ${E_{p}}^{-0.8}$.

Besides *E*_p_, another key term for *β* is *Z/N*_v._ In the most basic Bethe equation [28], *Z* plays an important role in the definition of electron density. However, in the latest TPP-2M formula, *Z* is not included because of an undiscovered relationship between the material-dependent parameters and the formula. Herein, through the powerful LASSO method, *Z/N*_v_ has been brought in the new formula as a major breakthrough. Figure 7(b) shows the linear relationship between *β* and *Z/N*_v_ except for alkaline metals. The important point is that *Z* has not been included in the TPP-2M formula yet, but *Z* was introduced to our TPP-LASSO formula by LASSO. Considering that *Z* is the total electron number, *N*_v_/*Z* can be considered the valence electron ratio of the total electron number, and *Z*/*N*_v_ is the reciprocal of it. Alkaline metals are seemingly ‘self-contained’ because they have another linear relationship. Despite alkaline metals being separated from other materials, there is still an obvious linear distribution.

**Figure 8.**
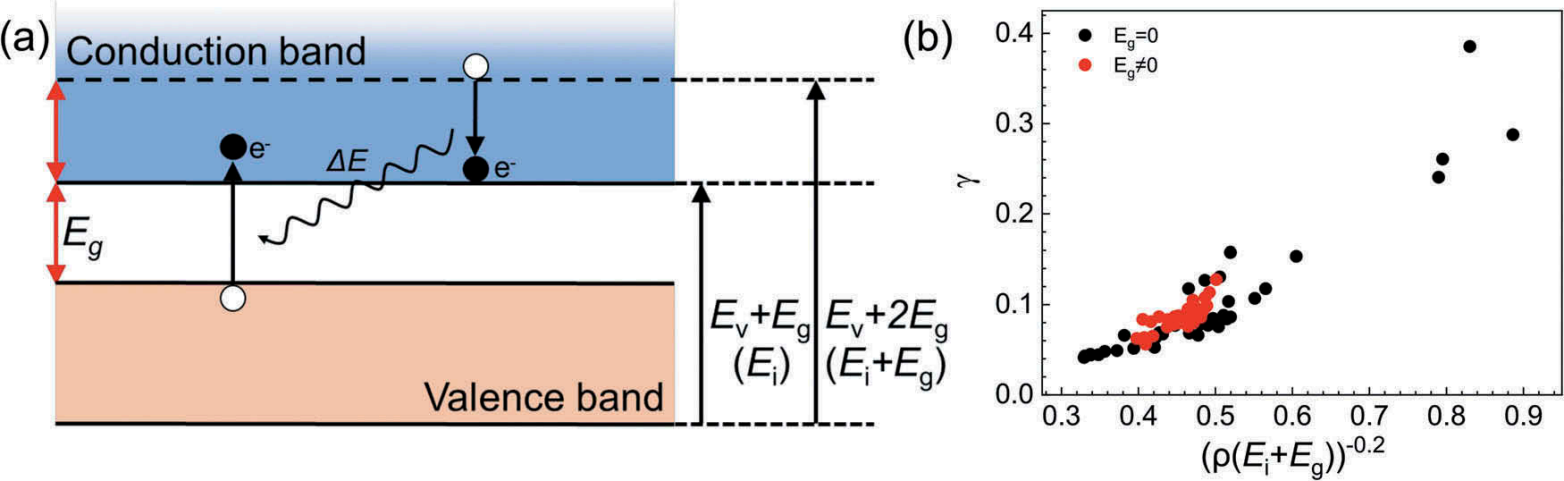
(a) Typical band structure and electron excitation process for insulators. Here *E*_g_ is the band gap energy and *E*_i_ is starting-point energy, which is the valence-band width plus the band gap energy for insulators. Suppose that there are two electrons: electron 1 at the energy of the valence band edge (*E*_v_) and electron 2 at the energy of *E*_g_ higher than the conduction band bottom (*E*_i_ + *E*_g_). Electron 2 gives energy to electron 1 and electron 1 is excited to the conduction band. There is therefore an energy restriction that electron 2 must be higher than *E*_i_ + *E*_g_ or else electron 2 will fall into the band gap after giving out energy, which is impossible. (b) Relationship between the principle descriptor [(*E*_i_ + *E*_g_)*ρ*]^−0.2^ and *γ*. The red (black) dots represent for materials with *E*_g_≠0 (*E*_g_ = 0).

Among the terms for *γ*, the most common term is [(*E*_i_ + *E*_g_)*ρ*]^−0.2^. This principle descriptor holds approximate physics meanings. In the case of metals, this term is simplified to associate with *E*_F_*ρ* and has a relatively obvious physics meaning related to the normalized Fermi energy, which is mainly affects secondary electron (SE) excitation in metals. As for semiconductors and insulators, the principle descriptor [(*E*_i_ + *E*_g_)*ρ*]^−0.2^ somewhat reveals the physics picture of SE excitation. Figure 8(a) shows a schematic diagram of the energy-band structure of a semiconductor or insulator with bandgap energy *E*_g_. When energetic electrons move inside an insulator, they may transfer all or part of their energy to electrons in the valence band, and then the electrons in the valence band can transfer across the band gap to the conduction band as a typical SE excitation process in a semiconductor or insulator. It is obvious that such SE excitation only occurs under the premise that the energy of the primary energetic electron must be above *E*_i_ + *E*_g_, i.e. *E*_v_ + 2*E*_g_ for insulators referring to the bottom of valence band, to excite an electron located at the top of the valence band across the band gap into the conduction band as the limiting case; at the same time, the primary energetic electron is still within the conduction band after losing energy. Correspondingly, the term (*E*_i_ + *E*_g_)*ρ* reflects the possibility of SE excitation of a semiconductor or insulator per unit volume. Therefore, LASSO selects this descriptor, suggesting that regardless of metals and insulators, SE excitations are strongly correlated to the electron inelastic scattering behavior.

Figure 8(b) shows the linear relationship between *γ* and [(*E*_i_ + *E*_g_)*ρ*]^−0.2^. In Figure 8(b), the red points represent materials in which *E*_g_≠0, in other words, insulators or semiconductors, and black points indicate metals. The red points share a common linear relationship with the black points, which means the descriptor [(*E*_i_ + *E*_g_)*ρ*]^−0.2^ holds for all kinds of materials when describing IMFPs. This is also evidence that this principle descriptor has a strong ability to generalize the IMFPs for all materials.

More than the isolated explanations for each descriptor, a more important physics picture is obtained by putting together the physics meaning of the descriptors. As known by physicists in the surface analysis field, the IMFP is a fundamental parameter describing the process of electron scattering when an energetic electron moves inside or near a material. In inelastic scattering, there are two main contributing excitations: single electron excitation and plasmon excitation. Reviewing the principle descriptors mentioned above, some relationships can be summarized: (1) As the principle descriptor of *γ*, [(*E*_i_ + *E*_g_)*ρ*]^−0.2^ reflects the SE excitation contributed by single electron excitations in various materials caused by different material band structures. (2) As the principle descriptor of *β, E*_p_, as its name suggests, occupies a very important position in the description of plasmon excitation in inelastic scattering [17]. In fact, plasmon excitation can be seen as a collective oscillation of valence electrons. Together with the single electron behavior in (1), it can be summarized that two main electron inelastic scattering behaviors caused by single electron excitation and plasmon excitation are included in the principle descriptors chosen by LASSO: *E*_p_ in *β* for collective behavior of valence electrons and [(*E*_i_ + *E*_g_)*ρ*]^−0.2^ in *γ* for individual behavior of valence electrons. Although the principle descriptors were produced completely digitally, they turned out to describe a meaningful physics picture.

## 4. Conclusions

On the basis of an existing database, we developed a new framework using ML to enhance the accuracy of an empirical formula and give a formula for the IMFP for an example. The parameters in the TPP-LASSO formula were thoroughly discussed using a Fano plot, and the LASSO algorithm was thus employed to select the combination of terms for these parameters. The LASSO algorithm demonstrated superior ability in reducing the number of terms without reducing the descriptive ability within an acceptable range. With the introduction of a system that analyzes importance, the balance of accuracy and convenience can also be adjusted easily. Besides improved accuracy, another important advantage of the framework is the ability of the framework to guide application or exploration. Herein, we provided data-driven evidence for the long-existing parameter *E*_p_ and innovatively introduced *Z* into the TPP-2M formula, which Tanuma et al. failed to do using the Bethe equation. A reasonable hypothesis of the connection between the band structure and IMFP is revealed by the framework’s selection of a major term. These contributions are strong evidence of the all-round ability of the new framework. Importantly, not limited to the application example of the IMFP here, the framework can easily be applied to other fields to determine a reliable empirical formula according to a specified database, while providing key descriptors to the field. The analysis is superior to traditional least-squares and remainder analyses in terms of accuracy, time taken, and convenience.

## Data Availability

All data generated and/or analyzed during this study are included in this article
