# Supplementary material for: Unveiling the principle descriptor for predicting the electron inelastic mean free path based on a machine learning framework
Source: Sci Technol Adv Mater. 2019 Nov 7;20(1):1090–102. doi: 10.1080/14686996.2019.1689785 (PMC6882444; doi:10.1080/14686996.2019.1689785)
Supplement: Supplemental Material [file TSTA_A_1689785_SM7030.pdf]

Supplementary materials: Unveiling the principle descriptor for predicting the electron inelastic mean free path based on a machine learning framework

Xun Liu[1,2,3], Zhufeng Hou[4], Dabao Lu[1,2,3], Bo Da[2,3*], Hideki Yoshikawa[2], Shigeo Tanuma[3], Yang Sun[5] and Zejun Ding[1#]

[1]*Hefei National Laboratory for Physical Sciences at Microscale and Department of Physics, University of Science and Technology of China, Hefei, Anhui 230026, People's Republic of China*

[2]*Research and Services Division of Materials Data and Integrated System, National Institute for Materials Science, 1-1 Namiki, Tsukuba, Ibaraki 305-0044, Japan*

[3]*Research Center for Advanced Measurement and Characterization, National Institute for Materials Science, 1-2-1 Sengen, Tsukuba, Ibaraki 305-0047, Japan*

[4]*State Key Laboratory of Structural Chemistry, Fujian Institute of Research on the Structure of Matter, Chinese Academy of Sciences, Fuzhou 350002, China*

[5]*Ames Laboratory, US Department of Energy, Ames, Iowa 50011, USA*

*DA.Bo@nims.go.jp

#zjding@ustc.edu.cn

1. Code example used in section "Establishment of the descriptor pool" and "Selecting principle terms with LASSO":

```
# python version: 3.6.3
import os
import numpy as np
import pandas as pd
from sklearn.linear_model import LassoCV
from time import clock

#paras.csv contains beta and gamma values derived from prototype formula and FPA-calculated IMFPs using the least squares algorithm
#features.csv contains features of each materials
```

```
fPath = 'paras.csv'
fPath_features = 'features.csv'
if os.path.exists(fPath)&os.path.exists(fPath_features):
        data0 = pd.read_csv(fPath,header=None,names=['beta','gamma'])
        features = pd.read_csv(fPath_features,header=None,index_col=
False,names=['Z','M','rou','Nv','Eg','Ei','R'])
else:
        print('No such file or directory!')

#material list
matid=['Li', 'Be', 'C(graphite)','C(diamond)','C(glassy)', 'Na', 'Mg',
            'Al', 'Si', 'K', 'Sc', 'Ti', 'V', 'Cr', 'Fe', 'Co', 'Ni', 'Cu',
            'Ge', 'Y', 'Nb', 'Mo', 'Ru', 'Rh', 'Pd', 'Ag', 'In', 'Sn','Cs',
            'Gd','Tb','Dy', 'Hf', 'Ta', 'W', 'Re', 'Os', 'Ir', 'Pt', 'Au', 'Bi',
            'AgBr', 'AgCl', 'AgI', 'Al2O3', 'AlAs', 'AlN', 'AlSb', 'cubic BN', 'hexagonal BN', 'CdS', 'CdSe',
'CdTe',
            'GaAs', 'GaN', 'GaP', 'GaSb', 'GaSe', 'InAs', 'InP', 'InSb', 'KBr', 'KCl', 'MgF2', 'MgO', 'NaCl',
'NbC0.712',
            'NbC0.844', 'NbC0.93', 'PbS', 'PbSe', 'PbTe', 'SiC', 'SiO2', 'SnTe', 'TiC0.7', 'TiC0.95', 'VC0.76',
'VC0.86',
            'Y3Al5O12', 'ZnS', 'ZnSe', 'ZnTe']

data0=np.array(data0)
features=np.array(features)
#label contains descriptor labels produced using our framework
label=['Z','M','rou','Nv','Eg','Ei','R']

#produce descriptors using our framework
#B
for i in [4,5]:
        for j in [4,5]:
                if i<j:
                        features=np.append(features,np.array([(features[:,i]+features[:,j])]).T,axis=1)
                        features=np.append(features,np.array([(features[:,i]-features[:,j])]).T,axis=1)
                        label.append('('+label[i]+'+'+label[j]+')')
                        label.append('|'+label[i]+'-'+label[j]+'|')
                        features=np.append(features,np.array([(features[:,i]**2+features[:,j]**2)]).T,axis=1)
                        features=np.append(features,np.array([(np.abs(features[:,i]**2-
features[:,j]**2))]).T,axis=1)
                        label.append('('+label[i]+'^2+'+label[j]+'^2)')
                        label.append('|'+label[i]+'^2-'+label[j]+'^2|')
for i in range(0,7):
        for j in range(0,7):
                if i<j:
```

```
            features=np.append(features,np.array([features[:,i]*features[:,j]]).T,axis=1)
            label.append('('+label[i]+'*'+label[j]+')')
        if (0 not in features[:,j])&(i!=j):
            features=np.append(features,np.array([features[:,i]/features[:,j]]).T,axis=1)
            label.append('('+label[i]+'/'+label[j]+')')
#C
t=len(label)
for i in range(0,7):
    for j in range(7,t):
        features=np.append(features,np.array([features[:,i]*features[:,j]]).T,axis=1)
        label.append('('+label[j]+'*'+label[i]+')')
        if 0 not in features[:,j]:
            features=np.append(features,np.array([features[:,i]/features[:,j]]).T,axis=1)
            label.append('('+label[i]+'/'+label[j]+')')
        if 0 not in features[:,i]:
            features=np.append(features,np.array([features[:,j]/features[:,i]]).T,axis=1)
            label.append('('+label[j]+'/'+label[i]+')')
#D
t=len(label)
for i in range(0,t):
    for j in [-9, -8, -7, -6, -5, -4, -3, -2, -1, 1, 2, 3, 4, 5, 6, 7, 8, 9]:
        if np.all(features[:,i]>0):
            features=np.append(features,np.array([(features[:,i]**(j*0.1))]).T,axis=1)
            label.append(label[i]+'^'+str(round(j*0.1,1)))

start = clock()

#LASSO for beta
data=data0[:,0]
lasso = LassoCV(alphas=None, copy_X=True, cv=5, fit_intercept=True,
    max_iter=99999, n_jobs=-1, normalize=True, positive=False,
    precompute='auto', random_state=None, selection='cyclic', tol=0.0001,
    verbose=False)
lasso.fit(features, data)
print('Alpha: ', lasso.alpha_)
print('Coefficients: ', lasso.coef_)
print('intercept: ', lasso.intercept_)
print('scores:',lasso.score(features, data))
#beta contains the coefficients of descriptors produced by LASSO (intercept included)
#beta0 contains the beta values regressed by LASSO
beta=np.append(lasso.coef_,lasso.intercept_)
beta0=lasso.predict(features)

#LASSO for gamma
```

```
data=data0[:,1]
lasso = LassoCV(alphas=None, copy_X=True, cv=5, fit_intercept=True,
    max_iter=99999, n_jobs=-1, normalize=True, positive=False,
    precompute='auto', random_state=None, selection='cyclic', tol=0.0001,
    verbose=False)
lasso.fit(features, data)
print('Alpha: ', lasso.alpha_)
print('Coefficients: ', lasso.coef_)
print('intercept: ', lasso.intercept_)
print('scores:',lasso.score(features, data))
#gamma contains the coefficients of descriptors produced by LASSO (intercept included)
#gamma0 contains the gamma values regressed by LASSO
gamma=np.append(lasso.coef_,lasso.intercept_)
gamma0=lasso.predict(features)

elapsed = (clock() - start)
print("time=",elapsed)
```
